# The Macrophage Response Is Driven by Mesenchymal Stem Cell-Mediated Metabolic Reprogramming

**DOI:** 10.3389/fimmu.2021.624746

**Published:** 2021-06-04

**Authors:** Noymar Luque-Campos, Felipe A. Bustamante-Barrientos, Carolina Pradenas, Cynthia García, María Jesús Araya, Candice Bohaud, Rafael Contreras-López, Roberto Elizondo-Vega, Farida Djouad, Patricia Luz-Crawford, Ana María Vega-Letter

**Affiliations:** ^1^ Laboratorio de Inmunología Celular y Molecular, Facultad de Medicina, Universidad de los Andes, Santiago, Chile; ^2^ Centro de Investigación e Innovación Biomédica, Universidad de los Andes, Santiago, Chile; ^3^ Programa de Doctorado en Biomedicina, Facultad de Medicina, Universidad de los Andes, Santiago, Chile; ^4^ Facultad de Ciencias, Universidad de Chile, Santiago, Chile; ^5^ Escuela de Biotecnología, Facultad de Ciencias, Universidad Mayor, Santiago, Chile; ^6^ IRMB, Univ Montpellier, INSERM, Montpellier, France; ^7^ Laboratorio de Biología Celular, Departamento de Biología Celular, Facultad de Ciencias Biológicas, Universidad de Concepción, Concepción, Chile; ^8^ Cells for Cells, Regenero, Las Condes, Santiago, Chile; ^9^ Laboratory of Nano-Regenerative Medicine, Facultad de Medicina, Universidad de los Andes, Santiago, Chile

**Keywords:** mesenchymal stem cells, metabolism, macrophages, cancer, tissue repair and regeneration, autoimmunity

## Abstract

Mesenchymal stem cells (MSCs) are multipotent adult stromal cells widely studied for their regenerative and immunomodulatory properties. They are capable of modulating macrophage plasticity depending on various microenvironmental signals. Current studies have shown that metabolic changes can also affect macrophage fate and function. Indeed, changes in the environment prompt phenotype change. Therefore, in this review, we will discuss how MSCs orchestrate macrophage’s metabolic plasticity and the impact on their function. An improved understanding of the crosstalk between macrophages and MSCs will improve our knowledge of MSC’s therapeutic potential in the context of inflammatory diseases, cancer, and tissue repair processes in which macrophages are pivotal.

## Introduction

Mesenchymal stem/stromal cells (MSCs) are multipotent cells with the capacity of differentiating into cells from the mesodermal tissue such as adipocytes, chondrocytes, and osteoblasts cells (1, 2). MSCs are heterogeneous stromal cells that exert pleiotropic effects, including inhibition of inflammation and apoptosis through their capacity to produce bioactive molecules. Moreover, MSCs are well described for their immunoregulatory properties since they can control cells from innate and adaptive immune systems (3). Therefore, MSC-based therapy has been under evaluation for the last 15 years due to these properties for several relevant diseases (3). MSCs actively interact with components of the innate immune system displaying mainly anti-inflammatory effects (4). Indeed, they can regulate a cells’ response from innate immune systems such as dendritic cells, natural killer cells, and macrophages. Currently, we know that MSCs can orchestrate pro-inflammatory conditions (5) and tissue repair through the regulation and promotion of macrophage polarization towards an anti-inflammatory phenotype expressing high levels of arginase 1 (Arg1) (6), metalloproteinase-9 (MMP-9) (7), and CXCR4/stromal cell-derived factor (SFD-1) axis (8). MSC’s total capacity to control macrophage fate and functions is beneficial for several diseases such as myocardial infarction (9), acute kidney ischemia (10), diabetes (11, 12), and arthritis (13). However, there is a significant gap between MSC-based clinical trials and the underlying mechanisms of interaction with other cells. Therefore, a better understanding of the underlying mechanisms of MSC-mediated regulatory functions in macrophages would improve their therapeutic potential. Recently, the role of cell metabolism in macrophage functions has been intensively investigated (14–19). The pivotal role of their metabolic status on their phenotype and different processes could be relevant for improving the therapeutic potential of MSCs in macrophage-mediated diseases. Recently, metabolism has been a widely studied field considering its role in cell fate and functions. In this context, the signals sensed from the external environment are crucial to coordinate the macrophage polarization towards either a pro- or anti-inflammatory phenotype and the consequential impact in the progression of some pathologies’ severity. Therefore, in this review, we synthesize macrophage response evidence driven by mesenchymal stem cell-mediated metabolic reprogramming and how this regulation will finally impact the progression of several inflammatory and autoimmune diseases.

## Macrophage Plasticity, and Function

### M1 and M2 Macrophage

In healthy individuals facing an injury or infection, there is a sequential inflammatory response ending in the resolution of inflammation and tissue repair (20). In this context, monocytes, found in the bone marrow, bloodstream, or spleen, play an imperative role (21). Wounds and infected sites recruit monocytes where they differentiate into different macrophage subsets, including pro-inflammatory macrophage. Mills et al. described this concept during the 2000s showing that macrophage could generate a CD4 T-helper 1 (Th1) response, while others induced a response from CD4 T-helper 2 (Th2) (22). Therefore, they assigned such names as M1-like and M2-like macrophage subtypes referring to pro-and anti-inflammatory macrophages, respectively. Polarization is the transition process from an M1-like towards an M2-like phenotype (23), associated with both activation and resolution of inflammation, respectively (22). In sum, M1-like macrophage can be defined as pro-inflammatory cells, responsible for initiating the immune response (24) and characterized by high levels of CD80, CD86, and MHCII, among others (25, 26). Recently, CD38, the transmembrane receptors Gpr18, and formyl-peptide receptor 2 (Fpr2) have been described to be specific for M1 macrophage (25–27). The production of interferon-*γ* (IFN-*γ*) due to tissue damage, the presence of pathogens or cytokines [such as macrophage and granulocyte colony-stimulating factor (GM-CSF) (28, 29)] by T lymphocytes (CD4 or CD8), natural killers (30, 31), or lipopolysaccharide (LPS) recognition expressed by pathogens, causes macrophage activation towards an M1 phenotype (32). In response, M1 macrophages increase the secretion of pro-inflammatory mediators such as nitric oxide (NO), tumor necrosis factor-α (TNF)-α, interleukin (IL)-6, IL-15, IL-12, IL-23, IL-1β (21), and additionally increase the ability to present antigens (29). M2-like macrophages, (also called alternatively activated macrophage) have an anti-inflammatory phenotype, relevant in immunity regarding the resolution of the inflammatory process, elimination of cell debris, promoting angiogenesis, regeneration, and wound healing of damaged tissue, parasitic helminths, tumor growth, and metastasis (33, 34). In general, M2 macrophages express high levels of CD206 and CD163 and low levels of CD80, CD86 (co-stimulatory molecules), and MHCII (35, 36). Several factors in the microenvironment induce macrophage polarization towards the M2 phenotype, including IL-4, IL-10, IL-13, IL-21, and IL-33 (37). M2 macrophages are generally characterized by an increased secretion of the anti-inflammatory cytokine IL-10 (38).

Likewise, upon stimulation using classic macrophage differentiation and activation (LPS or IFN-*γ*) or using alternative activation (IL-4 or IL-13), macrophage undergoes insightful metabolic reprogramming critical in activating their cellular mechanisms and successfully defying the infection during inflammation resolution (14, 15, 39). Therefore, we will discuss this sophisticated regulation of the metabolism on a macrophage’s fate and how this affects their functionality.

### Metabolism Regulation on M1 Macrophage Polarization: Enhanced Glycolysis and a “Broken” Krebs Cycle Support Their Phenotype and Function

**Figure 1:**
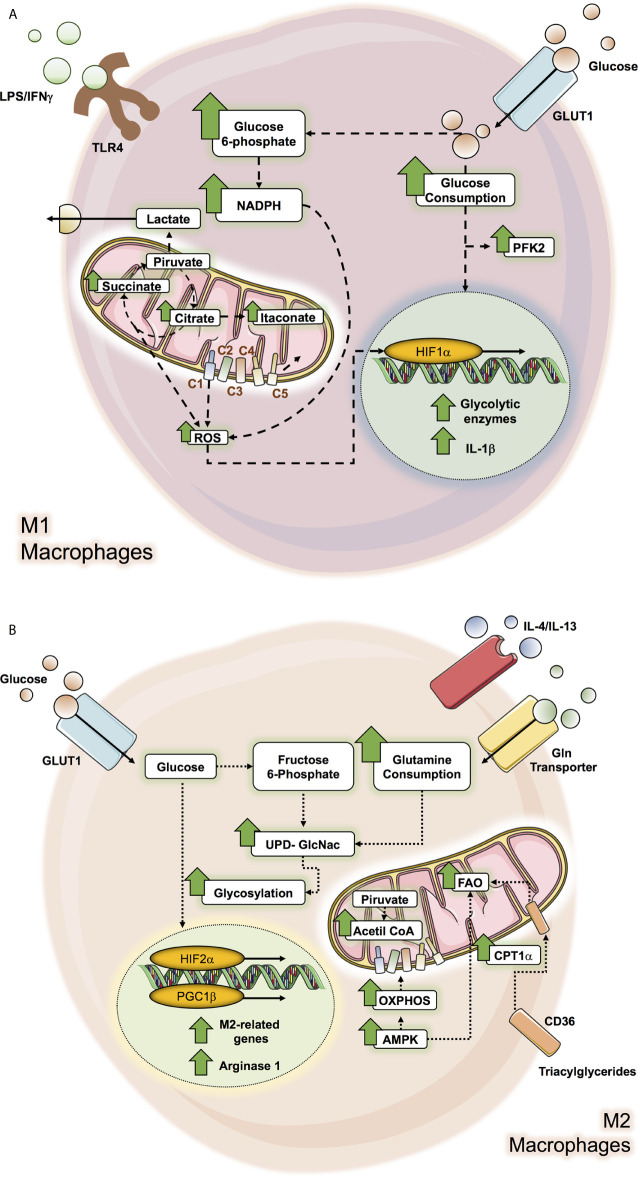
Metabolic pathways altered in M1 and M2 macrophage. macrophage metabolic pathways can be altered by microenvironment molecules as glucose, interleukines and LPS/IFN-γ according to the macrophage subtype polarization, where M1 macrophage **(A)** affected pathways correspond to glycolysis and Krebs cycle while in M2 macrophage **(B)**, the OXPHOS and the FAO pathways are the ones which can be altered.

A microenvironment enriched with TNF-α and IFN-*γ*, or with LPS, will induce monocytes to activate/differentiate into M1 macrophage (40). M1 activates their metabolism and antimicrobial activity through the nicotinamide adenine dinucleotide phosphate (NADPH) oxidase system, thereby producing reactive oxygen species (ROS) (41). However, high production of ROS could ultimately be detrimental to the body itself. Thus, this response’s inhibition occurs by increasing the amount of M2 macrophage, either by converting M1 macrophage or by recruiting pro-inflammatory monocytes that switch to an anti-inflammatory profile (42, 43). The presence of IL-4 and IL-13 promotes the M2 phenotype (44). There are variations within the M2 phenotype: M2a, M2b, and M2c, whose differences are related to each subtype’s activator molecules (45). The metabolic reprogramming of immune cells represents a new exciting field that has improved our understanding of immune cell and macrophage function. However, how the external signals provided by other cell types within the altered tissue can promote macrophage metabolic reprogramming has to be further investigated. Here, we summarize M1-like and M2-like macrophage’ main metabolic pathways, highlighting those that could be modulated by MSCs (**Figure 1**).

It has been well documented that M1 macrophages induced by IFN-*γ* and/or TLR ligands like LPS mainly exhibit dependency on the Warburg effect (aerobic glycolysis) by increasing glycolysis and lactate release (14, 15, 17, 39, 46–48). Inflammatory LPS-stimulated macrophages enhance glucose consumption by up-regulating the glucose transporter, GLUT1, to facilitate a rapid glucose uptake (49). Indeed, GLUT1 is the leading rate-limiting glucose transporter in pro-inflammatory macrophage, and its overexpression displays a hyperinflammatory state characterized by increased expression of inflammatory mediators (50). These increased pro-inflammatory environments could be reversed by inhibiting glycolysis by 2-DG, which blocks early steps in glycolysis (50). Notably, glycolytic metabolic adaptation in M1 macrophage results from the stabilization of the transcriptional factor hypoxia-inducible factor 1-*α* (HIF-1*α*) (50, 51). HIF-1*α*, a master regulator of glycolysis, mediates the transcriptional induction of many glycolytic enzymes (51). LPS signaling activates HIF-1*α*, positively regulating the pro-inflammatory cytokine IL-1β that binds directly to the IL-1β promoter and other HIF-dependent genes, including those encoding enzymes in glycolysis and GLUT1 (52). Likewise, several enzymes of the glycolytic route are up-regulated in M1 macrophage, such as an isoform of phosphofructokinase-2, u-PFK2, to potentiate the glycolytic flux (53, 54).

Despite glycolysis being relatively inefficient to produce adenosine triphosphate (ATP), it can provide intermediate factors for the biosynthesis of macromolecules. The main example is glucose-6-phosphate which can enter the pentose phosphate pathway (PPP), allowing the production of NADPH and ribose-5-phosphate. In this context, LPS is promoted in the up-regulation of macrophage of hexokinase activity along with augmented glucose-6-phosphate dehydrogenase (G6PD) expression and activity, correlating with increased pro-inflammatory cytokines such as IL-6, monocyte chemo-attractant protein-1 (MCP-1), nitric oxide synthase (iNOS), and TNF-α (55, 56). G6PD is the first rate-limiting enzyme of PPP; therefore it suggests a convergence of glucose flux through both the glycolytic and PPP pathways (56). Moreover, during the oxidative phase of PPP, the NADPH formed is then used by several enzymes, including the NADPH oxidase, which generates ROS. Increased ROS production in M1 macrophage is essential for their anti-pathogen activity (50, 56), while NADPH also acts as an antioxidant defense (57).

One of the main metabolic signatures of LPS-stimulated macrophage is determined by associated defects in the Krebs’s cycle activity (39, 51). The Krebs cycle catabolizes Acetyl-CoA, generated in glycolysis, through a series of enzymatic reactions (58). HIF-1*α* induces the expression of the enzyme pyruvate dehydrogenase kinase (PDK), which inactivates pyruvate dehydrogenase (PDH) therefore limiting pyruvate-derived acetyl-CoA to incorporate into the Krebs cycle (54, 59). To maintain redox potential in M1 macrophage, pyruvate is converted into lactate, thus restoring NAD+ and following the glycolytic pathway, a key point considering that the mitochondrial oxidative phosphorylation system (OXPHOS) is impaired in M1 macrophage (14, 15, 39, 47, 60, 61). The decrease of carbon flux towards the Krebs cycle in pro-inflammatory macrophage profoundly impacts pathways and metabolites generated by this cycle as a consequence of metabolic reprogramming towards a high glycolytic activated status (14, 15). Moreover, several researchers have revealed that the disruption of the Krebs cycle has deep implications in the macrophage inflammatory phenotype (18, 62). They promote oxidative phosphorylation in a “broken” Krebs cycle after citrate and after succinate (14, 62). For example, the first broken step occurs by a reduced expression of the enzyme isocitrate dehydrogenase (IDH), which produces *α*‐ketoglutarate (*α*‐KG) from isocitrate. This alteration leads to the accumulation of citrate. Citrate is converted into oxaloacetate and acetyl-CoA and its synthesis of pro-inflammatory regulators such as nitric oxide (NO), ROS, prostaglandins, among others, drives the pro-inflammatory properties in these cells (63). Citrate is exported by mitochondrial citrate carrier (CIC) towards the cytosol since it’s the only space where it could be metabolized, and its inhibition impacts the inflammatory mediators previously mentioned (63, 64). Accumulated citrate can be metabolized into itaconate (60, 65). The enzyme involved in this conversion is aconitate decarboxylase 1 (encoded by *Acod1*, also referred to as *Irg1*), and its elevation is consistent in M1 macrophage (60, 65). Several works have pointed out that itaconate is one of the main metabolites produced by M1 macrophage (59, 60, 65). Another reaction is represented by the impairment in the succinate dehydrogenase (SDH) function in M1 macrophage, which catabolizes succinate to fumarate, thus the second breakpoint drives the accumulation of succinate (60). Succinate acts as a pro-inflammatory signal that inhibits prolyl hydroxylases (PHDs), which finally stabilizes HIF-1*α*. HIF-1*α* induces the expression of pro-inflammatory cytokines such as IL-1β and genes dependent on HIF-1*α*, such as enzymes implicated in the glycolytic pathway, and thus impacts by enhancing the glycolysis process which is essential for inflammatory macrophage activation (51, 66, 67). This event favors the excess of succinate being transported out of the mitochondria, impairing the PHDs function and thus, HIF-1α activation (68). Another anaplerotic pathway activated in M1 macrophage is the aspartate‐argininosuccinate shunt supply, malate. Considering the dysfunctionality of SDH, it induces an increase in malate levels. The aspartate‐argininosuccinate shunts are coupled with the urea cycle, which is relevant for NO and IL-6 production and therefore for M1 macrophage function (60).

### Metabolism Regulation of M2 Macrophage Polarization: Enhanced OXPHOS and FAO Promote Their Phenotype and Anti-Inflammatory Effect

Several studies have pointed out that M2 macrophage do not show an increase in glycolytic activity and are not required for M2 differentiation (15, 39, 48). However, other studies have shown that following IL‐4 stimulation, M2 increases the use of glucose (46, 60, 69, 70). On the other hand, HIF-2*α* activation, another isoform of HIF, is primarily observed in M2 macrophage and induces the expression of Arg1, while inhibiting the production of NO (71). Moreover, M2 macrophages express PFKFB1, a particular isoform of PFKF. The PFKFB1 isoform has high activity, which transforms fructose‐2,6‐bisphosphate into fructose‐6‐phosphate much more efficiently, therefore diminishing glycolytic rate (54).

In contrast to M1, M2 macrophages exhibit an entire Krebs cycle that is coupled to OXPHOS activity (14, 72). The stimulation of IL-4-stimulated macrophage promotes the increase of Acetyl-CoA production, which is controlled by protein kinase B (Akt)-mammalian Target of Rapamycin (mTORC1) pathway that induces the enzyme Acyl, responsible for its production (70). Acetyl-CoA participates in epigenetic reprogramming of M2 macrophage through the fine regulation of a subset of M2 genes (70). Moreover, the inhibition of mitochondrial ATP synthase using oligomycin suppresses IL-4-stimulated M2 in terms of its surface markers, genes, and functions (70, 72). Under this context, glucose drives the Krebs cycle to mitochondrial respiration in M2 macrophage. Similarly, these inhibitions in M2 preclude the ability to decrease pro-inflammatory cytokines such as IL-6, TNF-α and IL-12, also, IL-4, but not LPS/IFN-*γ* induction in macrophage, induces an increase in cellular mitochondrial mass (73). Furthermore, opposing the high production of NO in M1 macrophage as part of several inflammatory functions, the production of NO in M2 macrophage is low, allowing OXPHOS to be sustained (16).

M2 macrophage possesses a high AMP-activated protein kinase (AMPK) activity (74), a widely conserved metabolic sensor of AMP/ATP and glycogen in mammalian cells, which is also pivotal for the induction of oxidative phosphorylation (OXPHOS) and fatty acid oxidation (FAO) (75). AMPK increases the expression of proteins associated with OXPHOS, including peroxisome proliferator-activated receptor (PPAR)-**γ** coactivator (PGC) 1**β** (73). PGC-1**β** coactivators intervene by linking mitochondrial biogenesis to biological processes that are associated with increased oxidative metabolism, a distinctive feature of this coactivator (76). Alternatively, macrophages activated by IL‐4 strongly induce PGC-1**β** expression in a signal transducer and activator of transcription-6 (STAT6)-dependent manner (73). PGC-1*β* coactivates the transcriptional functions of STAT6 (73), a critical factor that controls the genetic program for long-term macrophage activation (77) and promotes the maturation of the M2 phenotype to counterbalance excessive inflammation leading to enhanced tissue repair (78). The transgenic expression of the metabolic coactivator protein PGC-1*β* potentiates M2 macrophage’s activation, demonstrated by how RNAi knockdown of PGC‐1*β* impairs both metabolic and anti-inflammatory functions of IL-4-activated macrophage (73). Moreover, under IL-4 stimulation and the inhibition of OXPHOS, macrophages are incapable of producing LPS-induced TNF-α, IL‐12p40, and IL‐6 (73). This study supports a requirement of M2 macrophage for mitochondrial respiration and confers a pivotal role in PGC-1*β* to achieve their anti‐inflammatory functions. Taken together, these results spotlight that oxidative metabolism could be the acceptable bioenergetics status to perform the long-term activation, being PGC-1*β*/STAT6 axis necessary to couple the metabolic pathways and less-inflammatory immune functions of M2 phenotype.

M2 macrophages consume glutamine at high rates in the absence of glycolysis to supply OXPHOS (46, 79). Indeed, it has been well determined that glutamine-deprived M2 macrophages affect their polarization by decreasing CCL22 production, Irf4, Klf4, and Il4i1, pivotal molecules in these cells (60, 80). Remarkably, the deprivation of glutamine in M2 macrophage displays down-regulation of the Krebs cycle activity, since glutamine consumes OXPHOS (60). Glutamine could act at different levels in M2 macrophage; for example, glutamine provides a substrate for the Uridine-diphosphate-N-acetylglucosamine (UDP-GlcNAc) synthesis, which is a product of the hexosamine biosynthetic pathway. This pathway incorporates metabolites produced from nucleotide synthesis, glycolysis, glutamine, and Acetyl-CoA. In M2 macrophage, UDP-GlcNAc is vital for N-glycosylation, owing to the impact in their function to its inhibition, significantly inhibiting M2 activation markers expressed abundantly, including Relmα, CD206, and CD301 (60). Indeed, it is well recognized that M2 key markers (mannose-binding lectin receptor) are highly glycosylated (81). This data exposes a critical role of UDP-GlcNAc synthesis in the M2 polarization (60). Likewise, glutamine also plays a vital role in epigenetic regulation, since *α*-KG production from glutaminolysis is essential for M2 OXPHOS and FAO, affecting the demethylation of M2-specific marker genes. This induces PHD activity and thus inhibits HIF-1*α* expression (60, 82).

## MSCs Regulate the Metabolic Reprogramming of Macrophage Polarization

In macrophage, the transition from a pro-inflammatory (M1) to an anti-inflammatory/pro-regenerative (M2) phenotype, or *vice versa*, is marked by the modification of diverse metabolic pathways, such as (i) glycolysis, (ii) oxidative phosphorylation (OXPHOS), (iii) tricarboxylic acid cycle (or Krebs cycle), and (iv) fatty acid oxidation (FAO). We have discussed how metabolic reprogramming is an essential aspect of the balance between M1 and M2 macrophages. Their dynamics are controlled by the activity of intracellular signaling mediators activated by extracellular signals including cytokines, pathogens, and damage-associated molecular patterns. The strong inflammatory stimuli provided by a combination of IFN-*γ* and LPS drive towards an accelerated glycolytic metabolism (14, 15, 17, 39, 46–48) and to a ‘broken’ version of Krebs cycle, with concomitant accumulation of several metabolites such as succinate, citrate, and itaconate that sustain the inflammatory properties of M1 macrophage (18, 39, 51). On the other hand, cytokines such as IL-4 and IL-13 promote an inflammatory M2 phenotype demonstrated by enhanced OXPHOS and FAO (19, 47) and increased glutamine metabolism (46, 60, 79). All this evidence points out to a clear stage on how the regulation of metabolic reprogramming in macrophage is an interesting field to explore for its therapeutic potential. In this context, it has been extensively reviewed that MSCs affect macrophage’s fate through several immunosuppressive molecules (32). Furthermore, we and others have been discussing data supporting the insight about how metabolism rewiring sophisticatedly regulates the macrophage’s fate. Therefore, it is reasonable to think that MSCs could act beyond polarization towards an anti-inflammatory M2 macrophage phenotype by remodeling their metabolic programs and thus, their function. Indeed, besides the relevance of this incoming review, there are a couple of reports about how MSCs could metabolically manipulate macrophage’s fate. These studies lead us to the following discussions, allowing us to draw a new perspective to investigate. In the following paragraphs, we develop essential MSCs molecules in macrophage modulation, summarized in **Table 1**.

**Table 1:** Summary of the articles that demonstrated the metabolic crosstalk between MSCs and macrophages.

Origin	Context and/or Condition	MSC-mediated immunomodulatory mechanism	Macrophages effect		Physiologycal Effect	Reference
			Molecular	Metabolic		
Human-Bone Marrow (BM)-MSC	Pathogen clearance	PEG2 dependant mechanism	Inhibition of CD80, CD86 while increased CD206 expression. Also inhibition of VEGF, and TNFα production and increase IL-10 production	Induction of an OXPHOS metabolism	Increased phagocytic activity of macrophages; Increased pathogen clearance	(83)
Human-BM-MSC-derived exosomes	Exosomes isolated from MSC under inflammatory and hypoxia conditions	Several metabolites with known suppressive activity on Macrophages	Not determined	Not determined	No determined	(84)
Murine-Adipose-derived MSC (ASC)-conditioned Media	Culture of murine-derived macrophages with the conditioned media of ASC	Not determined Just associated to ASC secretome	Increased production in ARG1 and IL10. Inhibition of TNFα production	increased PPARy and modulation of p-AKT, p-mTOR to increase OXHPOS	Increased lipids droplet neogenesis on M2-generated macrophages	(85)
Human-Umbilical-Cord (UC) MSC	Culture of UC-MSC monocytes induced to differentiated into dendritic cells	Lactate production	Decreased CD80/CD86 expression, increased IL10 and TGFb1 production	Induction of OXPHOS-related genes and induce a M2-macrophage expression signature	Generation of M2- macrophages	(86)
Murine-Bone Marrow (BM)-MSC	Sirtrulin 1-overexpressing MSC	IFN-γ and CXCL-10 production	Increased iNOS expression	Not determined	Macrophages recruitment and decreased prostate tumor growth.	(87)

**Figure 2:**
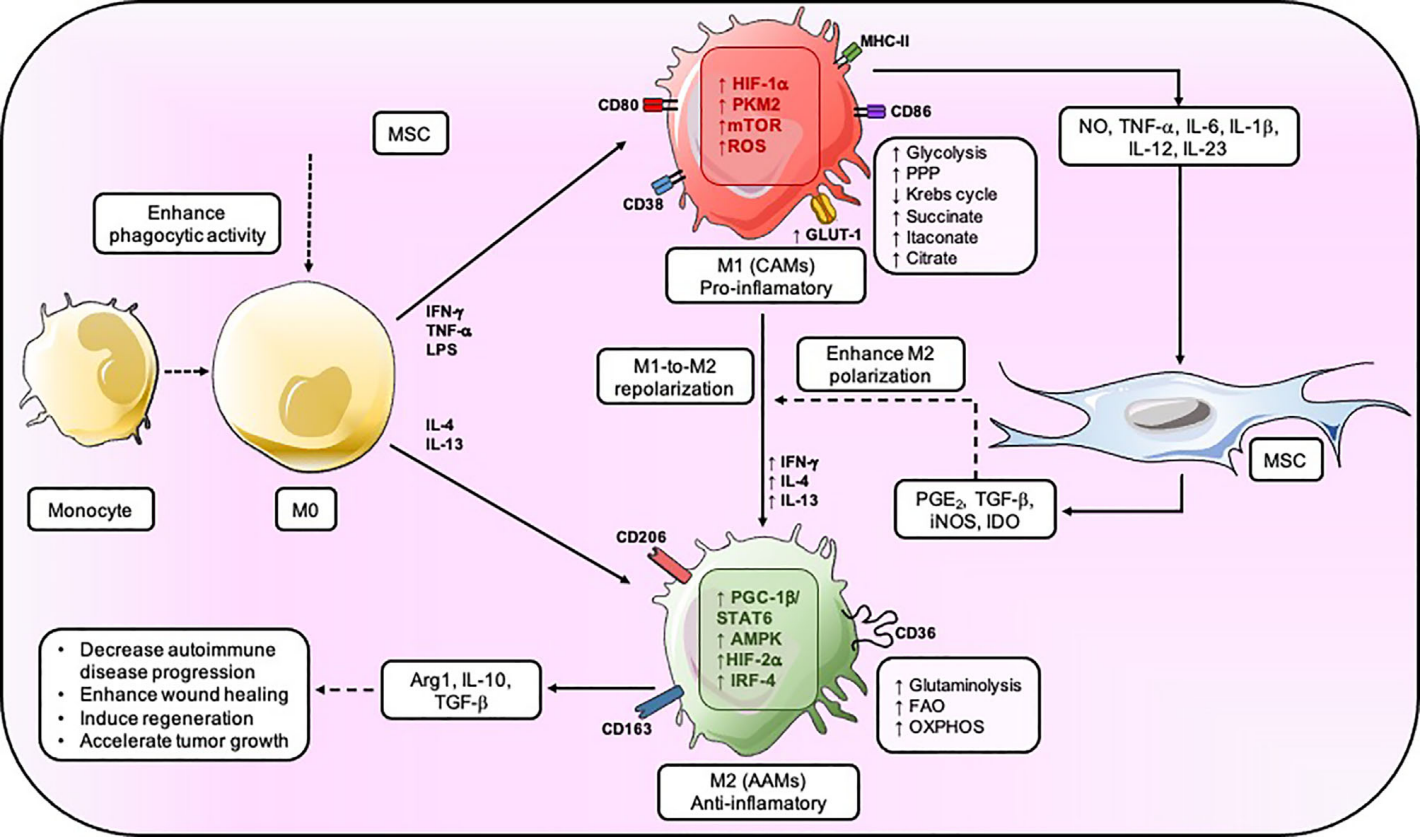
MSC regulates the metabolic fate of macrophage. MSC enhances the phagocytic activity of M0, but the inflammation generated by the M1 results in an increase in glycolysis, PPP, and a decrease in Krebs’s cycle. MSC provokes PGE_2_, TGF-β, INOS, and IDO to induce M2-like macrophage that mainly have an OXPHOS metabolic status and secrete IL-10, Arg1, and TGF–β which will reduce autoimmune disease progression, enhance wound healing, and accelerate tumor growth. CAMs, classically activated macrophage; AAM. alternatively activated macrophage.

MSCs can modulate the bioenergetic status driving macrophage’s polarization depending on the local microenvironment. For example, bone marrow-derived-MSCs (BM-MSCs) potentiate the macrophage-mediated clearance of pathogens such as *Salmonella* sp. by increasing their respiratory bursts *in vitro* (83). The MSC-mediated metabolic switch on macrophage is characterized by an increased expression of inducible NADPH oxidase subunits, such as PHOX p22 and PHOX p47, and subsequently elevated ROS total levels (83, 88); while the up-regulation of the SOD2 expression represents a compensatory antioxidant mechanism (83). It is important to mention that the amount of ROS being generated has a pivotal role in the metabolic differences of M1 and M2 macrophages and, therefore, in their function. In M2 macrophage, mitochondrial respiration is adequate, leading to low amounts of ROS. Conversely, M1 macrophage undergo OXPHOS dysfunction, succinate accumulation, and high mitochondrial membrane potential, leading to high ROS production (60, 67, 89, 90). Enhanced ROS production following succinate oxidation alters HIF-1*α* and thus IL-1β expression (89, 90). While mitochondrial ROS production principally occurs in complexes I and III, it has been shown that, in M1 macrophage, ROS is mainly generated by reverse electron transport (RET) in complex I rather than complex III and is required to counteract bacterial infection (52, 61, 89) effectively. The inhibition of complex I by either rotenone or metformin decreases LPS-induced ROS and, in turn, reduces IL-1β expression (67, 91, 92). Thus, mitochondrial ROS production generated by RET is pivotal for pro-inflammatory macrophage function, including phagocytosis, bacterial killing, and sustained inflammation through the stabilization of HIF-1*α*, giving way to the secretion of a potent inflammatory mediator: IL-1β (60, 67, 91, 93). Indeed, several studies have reported that MSCs enhance the phagocytic activity of macrophage by altering their differentiation from monocytes into inflammatory M1 macrophage, measured as an increase in the expression of typical markers including CD68, CD14, and CD11b (83, 88, 94–96). Besides, co-culture experiments reveal that BM-MSCs induce the down-regulation of indicator markers of antigen-presenting cells in macrophage’s (APC) functions, including CD80/CD86, CD50, CD54, MHCI/II; as well as a reduction of pro-inflammatory cytokines, such as IL-6, IL-12 and TNF-α (83). MSCs also produce a decrease in M1 macrophage-exerted endothelial injury function (a typical function of M1 phenotype), possibly due to the increase of MSC-secreted VEGF as a protective effect (83) (**Figure 2**). It is possible that MSCs modify macrophage activation in response to bacterial infection through a mechanism that includes an increase of mitochondrial ROS production as a consequence of the accumulation of succinate, by the stabilization of HIF-1*α*, which enhances glycolysis. However, further studies will be necessary to improve our knowledge on the macrophage metabolic reprogramming mediated by MSC, considering the succinate/ROS axis as a critical pathway for the macrophage pro-inflammatory phenotype.

One of the key metabolic sensors carrying the macrophage polarization/function is AMPK and mTOR, which integrate the external signals given by nutrient availability/deficit (97, 98). AMPK is a conserved serine/threonine kinase that regulates energy homeostasis and metabolic stress. When the cellular AMP/ATP ratio is high (low cellular energy charge), AMPK is activated, switching off ATP-consuming anabolic pathways and switching on ATP-producing catabolic pathways. AMPK acts by inhibiting anabolic pathways, and in parallel inducing catabolic pathways, such as FAO. AMPK also regulates carnitine palmitoyltransferase 1*α* (CPT1*α*), a facilitator of long‐chain fatty acid transportation through the outer mitochondrial membrane (75), thus regulating the uptake of fatty acids. Furthermore, AMPK is a coordinator in the production of pro-inflammatory cytokines by the acetylation of nuclear factor *κ*B (NF*κ*B) (99) through Sirtuin1, a downstream target of p-AMPK (100). Thus, AMPK is an inhibitor of the pro-inflammatory process initiation (100). Indeed, it has been well demonstrated that AMPK and mTOR are critical players for the function of M2-like macrophage through the FAO metabolism (19). AMPK activity is enhanced in IL-10 or TGF-*β*-stimulated macrophage (74). In contrast, LPS stimulation in macrophage results in a significant reduction of p-AMPK, and also AMPK ablation promotes the increase of TNF-α, IL-6 and cyclooxygenase 2 (COX-2) expression (74). CPT1α is also relevant for M2 polarization, owing to their inhibition to etomoxir decrease in the induction of arginase activity (73). AMPK activation and CPT1α expression in macrophage under different stimuli (pro- or anti-inflammatory) could be altered, promoting macrophage polarization towards an anti-inflammatory phenotype (74, 101). However, under IL-4 and colony-stimulating factor (M-CSF) activation (an essential factor in macrophage growth and survival) in macrophage, mTORC1 or mTORC2 signaling promotes glucose metabolism (69, 70). In this case, interferon-regulatory factor-4 (IRF-4), an inductor of a set of M2-specific marker genes such as arginase 1, mannose receptor, FIZZ1 and Ym1 (80), in turn, acts as an upstream signal for mTORC2’s activation playing a role in glucose metabolism that supports M2 activation and polarization (69). The inhibition of mTORC2 affects M2 macrophage activation and loss of capacity to battle helminth infection, one of the main protective responses exerted by these cells against parasitic infection (69). mTOR is important in supporting the glycolytic metabolism of M2 macrophage and their function through the expression of some of their markers. However, mTOR’s role is controversial on M1-like and M2-like macrophage. Indeed, it has been reported that mTOR’s function depends on the context, owing to the negative regulator of mTORC1, *Tsc1*, which can repress the M2-like macrophage phenotype while enhancing the activation of M1-like macrophage (70, 79). Based on this, MSCs influence the metabolic program and thus, the phenotype of M1 and M2 macrophage is already activated. MSCs co-cultured with IL-4-stimulated macrophage enhance p-AMPK and Sirtuin1 expression level but also decreases p-mTOR (83). At the same time, BM-MSCs provoke an increase of genes related to M2 phenotypes in IL-4-stimulated macrophage, such as anti-inflammatory cytokines IL-10 and TGF-β, scavenger receptors including CD205, CD206, CD163, CD36, and DC-SIGN (83). MSCs not only increase typical markers and some of the signature metabolic molecules of the M2 phenotype but also induce an anti-inflammatory M2 phenotype in M1 macrophage. MSCs co-cultured with LPS/IFN-γ-stimulated macrophage affect the glycolytic status of these cells by decreasing the genetic expression of GLUT1 and hexokinase 2 (HK2), and the phosphorylation of mTOR (Ser2448), which are metabolic features of M1 macrophage. In parallel, the genetic expression of CPT1α, as well as AMP-activated protein kinase (AMPK) phosphorylated (p-AMPK) (Thr172 [α2]) is increased in macrophage in contact with MSCs (83). Besides, BM-MSCs induce in LPS/IFN-γ-stimulated primary human macrophage the up-regulation of transcripts associated with M2 phenotype and simultaneously down-regulates the expression of some classical transcripts related to M1 macrophage (83). Under this context, MSCs induce an FAO metabolism on both M1 and M2 macrophage by the AMPK-mTOR axis. Also, MSCs enhance interferon-regulatory factor-4 (IRF-4) expression levels (100), which participate in M2 macrophage polarization by inducing Arg1, mannose receptor, Ym1, and FIZZ1 expression (102). MSCs could improve the resistance during helminth infection by increasing IRF4-mTORC2 axis activation and stabilizing the genes that drive M2 macrophage polarization. However, it has not yet been evaluated.

Furthermore, mTOR also participates in the modulation of lipid metabolism in macrophage through PPARγ, which is one of the main molecules regulating the expression of genes that control FAO (103). In macrophage, the pharmacological inhibition of lipid metabolism either by blocking the activity of PPARγ and mTOR leads to abnormal lipid droplet formation (85, 104). The source of the fatty acid that fuels FAO in M2 macrophage is mainly triacylglycerol. The uptake of triacylglycerol is facilitated through the CD36 receptor, which is implicated in the endocytosis of triacylglycerol-rich lipoprotein particles (105, 106). Lipolysis of triacylglycerol by lysosomal acid lipase (LAL) is crucial in providing the fatty acids that fuel OXPHOS. Thus, both increase OXPHOS metabolism and spare respiratory capacity (SRC). This event leads to the expression of genes and the long-term survival rate that sustains the M2 macrophage phenotype (107). The inhibition of lipolysis consistently reduces M2 macrophage activation and counteracts its response to challenge the infection caused by helminths (107). Notably, the expression of enzymes important for FAO, including acyl CoA dehydrogenase and enoyl CoA hydratase, is significantly enhanced by IL-4 (73). MSCs-produced immunosuppressive molecules can affect the bioenergetic status associated with lipids metabolism in macrophage. The conditioned medium obtained from adipose-derived MSCs (ASC) enhances the expression and activity of Arg1 in macrophage, favoring its transition towards the M2 phenotype (85). Furthermore, after being exposed to an ASC-derived conditioned medium, macrophage exhibited lipid droplet formation and thus PGE_2_ secretion through the up-regulation of COX-2 and phospholipases cPLA2-α (i.e., enzymes related to eicosanoids production) (85). The ASC-derived conditioned medium rescues the lipid metabolism of macrophage by promoting an increase of PPARγ expression, p-AKT (Ser473) and p-mTOR (Ser2448). Therefore, MSCs promote an enhanced FAO metabolic status and inhibition of the glycolytic metabolism in macrophage, features of anti-inflammatory M2 macrophage in detrimental pro-inflammatory M1 macrophage, respectively (**Figure 2**). In turn, these metabolic reprogramming processes are driven by MSCs through the tight regulation of molecules such as AMPK, mTOR, Sirtuin1, either at the mRNA or protein levels. MSCs possess the ability to skew already active M1 pro-inflammatory into anti-inflammatory M2 macrophage to resolve the hyper-inflammatory state shifting its metabolic reprogramming (83), which underlay pivotal mechanisms in macrophage’s fate decision, as we previously mentioned.

Considering that MSC-secreted immunosuppressive molecules could be involved in M2 macrophage’s metabolic switch, the inhibition of the enzyme COX-2 that catalyzes the conversion of arachidonic acid into PGE_2_ has proven to recover the metabolic profile of M1 macrophage. This recovery is characterized by the increase of the GLUT1 expression and TNF-α secretion, as well as the down-regulation of CPT1α, p-AMPK, and Sirtuin-1 on macrophage. Therefore, MSC-secreted PGE_2_ could be a candidate involved in the macrophage polarization by modulating the metabolic program in the interface between pro-inflammatory and anti-inflammatory macrophage (83). PGE_2_ signaling drives the migration of M2 macrophage to the wound healing area (108). However, how MSCs-derived PGE_2_ could affect the metabolic program of macrophage remains to be elucidated. On the other hand, it has been reported that MSCs promote an anti-inflammatory M2-like phenotype from monocytes undergoing differentiation into dendritic cells (DC), which is mediated by MSC through the release of lactate (86). The inhibition of lactate production in umbilical cord-derived MSCs (UC-MSCs) was abrogated with oxamic acid, an inhibitor of the enzyme lactate dehydrogenase, consequently resulting in the decrease of the expression of typical M2 genes including CD14, CD16, CD68, IL-10, and a high expression of CD1a (86). Furthermore, the conditioned medium of MSCs induces a decrease in mitochondrial biomass and an increase in spare respiratory capacity in monocytes differentiated towards DC (86). However, whether MSCs-derived lactate might be affecting the metabolism of macrophage remains unknown. In addition to classic immunosuppressive molecules secreted by MSCs, a novel mechanism of immunosuppression involving the secretion of extracellular vesicles (EVs) has been recently reported. EVs represent a novel path of cell-to-cell communication given by the release of vesicles ranging between 30-150 nm, which has moved into the center of attention because of their biological effects in receptor cells (86, 109). Lipids, proteins, and nucleic acids (including mRNAs, miRNAs, and other non-coding RNAs) are the main components of the exosome cargo whose profile content largely depends on the function and metabolic state of their parent cells since the EVs-cargo represents the contents of their cell’s origin (110). For example, it has been reported that miRNAs can modulate macrophage metabolism (111, 112). A recent study performed by Showalter et al. reported that MSCs-derived exosomes previously cultured under serum deprivation and hypoxic conditions carry several metabolites (84). MSCs-derived exosomes incorporate 21 different metabolites, including glutamine, adenosine, arginine, isoleucine, UDP-N-acetylglucosamine, 5’-deoxy-5’-methylthioadenosine, aspartic acid, palmitic acid, cholesterol, and nicotinamide (84). In this context, it has been well demonstrated that these metabolites are altered during immune cell activation, being directly linked to the functions they exert (47). Therefore, these MSCs-secreted metabolites could be incorporated by macrophage and driven to an anti-inflammatory phenotype, but studies to corroborate these observations are still needed. Thus, how MSCs-derived EVs containing miRNA or metabolites could modify the macrophage’s bioenergetic status is an interesting and unexplored field. This might improve our understanding of which MSCs-secreted immunosuppressive molecules might be modulating the role of macrophage in inflammatory diseases or during inflammation resolution beyond its bioenergetic status. In summary, the aforementioned data supports a new interesting field to explore and further study how the bioenergetic status of macrophage could be one of the underlying mechanisms following advantageous uses of MSCs under inflammatory microenvironments, by repolarizing them from an M1 to an M2 macrophage phenotype mainly through the enhancement of their OXHOS and FAO metabolism.

### The Relevance of Cellular Metabolism on MSCs-Macrophage Interaction

It is strongly believed that MSC mainly interacts with macrophages by paracrine action, however, it has been mentioned that these two types of cells can interact in other ways. It has been described that macrophages can interact with MSC through phagocytosis, which leads to their polarization into an M2 phenotype due to the secretion of COX-1, PGE_2_, and indoleamine 2,3-dioxygenase by MSC, that induced macrophage TGF-ß, IL-10, and Arg-1 expression (113–115). Even more, it has been shown that when macrophages phagocytose apoptotic MSC (emulating the conditions of injected MSC *in vivo*), it induces a signaling cascade in the macrophage which leads to the downregulation of TNF-α, IL-6 and NO pathways, suggesting a switch towards an OXPHOS metabolism, and the upregulation of CD163 and CD206, the characteristic surface markers for M2 macrophages (113, 116).

This data demonstrated that the interaction between macrophages and MSC goes beyond the paracrine activity of MSC, but that even apoptotic MSC has a strong and beneficial therapeutic effect on macrophages. As proposed by Stevens et al. (2020), apoptotic MSC could release mitochondria, which could significantly affect the activation of intracellular metabolic pathways, inducing an OXPHOS metabolism on macrophages, thus prompting a metabolic reprogramming into an M2 phenotype (115).

As mentioned before, MSC can dynamically interact with macrophage, being able to modulate their metabolic status. Therefore, in line with the previously mentioned, in the next section, we will focus on the metabolic crosstalk between MSC and macrophage which result in the generation of MSC-educated macrophage and their potential role in (a) cancer, (b) wound healing, (c) autoimmunity, and (d) regeneration.

#### a) Cancer

Cancer is one of the most lethal diseases of the last couple of decades and can arise from any organ or body structure. Tumors are composed of cells that have lost the ability to stop growing. A critical event in the genesis of cancer is the inability of immune cells of the body to identify and destroy newly formed cancer cells when their number is low (117).

Among the vast diversity of macrophage phenotypes, tumor-associated macrophage (TAMs) are macrophage that infiltrates solid tumors and are the dominant type of myeloid cells in cancer (25). These macrophages can de-differentiate into and from myeloid-derived suppressor cells (MDSCs) (118) and express genes usually associated with alternatively activated M2-like macrophage, like Arg1, mannose receptor C-type 1 (Mrc1), and others (25).

Although TAMs are mostly identified as M2-like macrophage, the characterization of these macrophages is more complex. Recently, it has been reported that TAMs are located in tumors that respond to smooth metabolic gradients that range from oxygen-rich vascular external regions to ischemic regions, where there is a lactic acid accumulation and nutrient deprivation (25, 119). In this context, because there is a combination of inflammatory and anti-inflammatory signals, such as TNF-α and IL-13, it results in a dynamic phenotype that escapes the usual macrophage classification. For example, perivascular TAMs express the M2 characteristic molecule Mrc1, but they lack the Arg1 marker. Conversely, the combination of hypoxia and lactate in distal areas of the vascular network directly activates mitogen-activated protein kinase (MAPK)/Ras-dependent extracellular signal-regulated kinase (ERK) signaling *via* proto-oncogene serine/threonine-protein kinase **(**cRaf), possibly through NDRG family member 3 (NDRG3), resulting in the co-expression of Arg1 and nitric oxide synthase 2 (Nos2), stereotypical M2 and M1 markers, respectively (119).

Due to the above, TAMs can acquire an anti-inflammatory role in tumors, where they have been shown to secrete pro-tumor signals (120), recruit the anti-inflammatory regulatory T cells (121) and dampen the T cell response (122). Because of that, TAMs play an important role in tumor growth, invasion, and metastasis (123). Furthermore, experimental studies indicate that increased numbers of TAMs are correlated with drug-resistance (124, 125) underlying therapy failure and poor prognosis of cancer (126, 127), as well as a reduced macrophage infiltration into the tumor, has been associated with diminished tumor growth (128). For example, Hughes et al. reported that M2 subpopulations of TAMs accumulate around blood vessels in tumors after chemotherapy, where they promote tumor revascularization and relapse (129).

Since the functions of TAMs largely depend on their accumulation and activation in tumor tissues, TAM-targeted antitumor approaches are mainly based on inhibiting macrophage recruitment, suppressing TAM survival, enhancing M1 tumoricidal activity of TAMs, and blocking M2 tumor-promoting activity of TAMs (124). According to this, the repolarization of TAMs into M1-like macrophage has successfully produced antitumoral responses in preclinical murine models (130).

In this context, MSCs have been shown to promote tumor progression by inhibiting the release of pro-inflammatory cytokines and increasing the generation of M2-like macrophage through the secretion of a variety of immunomodulatory molecules such as PGE_2_, IL1RA, TGF-β, and IL-8 (32, 131). The M2 polarization is produced by an increase in the expression of Arg1 and IL10, and by a decrease in the expression of IL1-β and TNF-α (132) (**Figure 2**). Interestingly, the tumor regulation exerted by MSCs appears to be modulated by the tumor itself. Pelizzo et al. characterized MSCs isolated and expanded from tumor tissues in pediatric patients diagnosed with neuroblastomas (NB-MSCs). Cell cycle analysis showed that MSCs had a higher number of cells in the G0-G1 phase compared to MSCs from healthy donors, thus supporting the essential role of MSCs in regulating cancer dormancy (19). Also, transcriptomic profiling results indicated that NB-MSCs were enriched with EMT genes, key in the initiation of metastasis (133). Analogously, the characterization of MSCs from patients with myeloproliferative neoplasms shows the lower median expression of CD146, a higher percentage of nestin-expressing MSCs, lower proliferative potential, reduced osteogenic differentiation capacity, and lower capacity to support long-term hematopoiesis *in vitro* than MSCs from healthy donors (134).

It has been shown that MSCs can exert an immunomodulatory effect on macrophage *via* cell-to-cell contact and paracrine actions (132). Thus, MSC–derived exosomes, which contain TGF-β, C1q, and semaphorins can also induce the differentiation of MDSCs into M2-polarized macrophage at tumor beds by driving Programmed Death-ligand 1 (PD-L1) overexpression and by inducing differentiation of macrophage with enhanced L-Arginase activity and IL-10 secretion (135). Analogously, MSC–conditioned medium increased mRNA and protein levels of Arg-1, CD206, and Ym1 expressions in macrophage (136). Also, it has been reported that malignant tumor cells can recruit MSC from surrounding tissue or the circulation, mediated *via* PDGF-β receptors on MSCs, and stimulate the angiogenesis process, resulting in tumor progression and metastasis (132).

Interestingly, in contrast to the tumor-promoting effect of MSCs described previously, MSCs that over-express Sirtuin 1 (MSC-Sirt1) can inhibit prostate cancer tumor growth by inducing IFN-γ production *in vivo*. IFN-γ activates macrophage and induces them to produce NO by iNOS, resulting in their increased tumoricidal activity (87). Therefore, the use of MSCs-Sirt1 is presented as a therapeutic option and opens the possibility of finding other regulatory proteins capable of reversing the immunomodulation exerted by MSCs in the tumor context.

#### b) Wound Healing

Wound healing definition refers to a superficial, epithelial or deep level of damage to nerves and muscles in the normal anatomical function-associated structure of the tissue (137). The healing process consists of a series of steps mentioned by A. Rivera and J. Spencer as hemostasis (clot formation), inflammation, proliferation or granulation, and matrix formation or remodeling, including an initial bleeding and coagulation process (137, 138). After the clot is formed, associated with the microenvironment consisting of IL-1s, TNFs, TGFs, PF4s, etc., monocytes migrate to the damaged zone. It is after a period of 48 to 96 hours that these monocytes differentiate into macrophage (139). However, the presence of other cells can affect the metabolism of macrophage, which is the case of MSCs. The interaction between MSCs and macrophage affects the IL-10 secretion on macrophage due to their production of PGE_2_, a prostaglandin that contributes to the M2 macrophage recruitment during wound healing (108). PGE_2_ binds to the prostaglandin E_2_ receptor-2 (EP2) and EP4 receptor expressed by macrophage, who has also been associated with the inflammation modulation by the cAMP/PKA signaling pathway in macrophage (5, 83, 108, 140, 141). This PGE_2_ interaction with macrophage through EP2 and EP4 receptors leads to adenylate cyclase activation and an increase in cAMP levels, thus activating the PKA pathway. In this cAMP/PKA pathway, there takes place the phosphorylation of cAMP-responsive element-binding (CREB) (Serr113), an important factor in macrophage in a wound healing context, increasing the transcription of C/EBP-β. This C/EBP-β promotes the expression of Arg1, Mrc1, and IL-10, the latter being an anti-inflammatory cytokine that blocks the function of the M1 macrophage (142–144). This mechanism could be the explanation of how MSCs are capable of modulating the metabolism of macrophage phenotype from M1 to M2 polarization in a wound healing context, through the PGE_2_ production.

Moreover, this modulation between MSCs and macrophage can also occur through the secretion of TNF-stimulated gene 6 (TSG-6) by MSCs, a protein that has shown to inhibit the release of TNF-α (145). TSG-6 leads to the polarization of M1 towards M2 macrophage by accelerating the wound healing process (146). TSG-6 also activates STAT1 and STAT3 while suppressing TLR4/NF-kB on macrophage (147). In macrophage, STAT1 is associated with the modulation of gene expression in proteins of glycolysis, OXPHOS, and the citrate cycle (148). Also, STAT3 is involved in the OXPHOs metabolism, as well as decreasing ROS production (149). It has been reported that STAT3 deletion causes a reduction in the activity of complexes I and II (149). These results suggest that MSCs could be favoring an OXPHOS metabolism in macrophage and, in turn, inducing M2 polarization through TSG-6 secretion.

Likewise, MSCs exposed to IFN-γ and TNF-α promote the up-regulation of IDO secreted by MSC (150), which in turn promote M2 anti-inflammatory macrophage polarization from monocytes (151). Previous studies have also indicated the possibility that the MSCs-macrophage interaction (in a wound healing context) leads to a higher expression of CD206, i.e. acquiring the M2 phenotype (145). This CD206 receptor, also known as the mannose receptor, regulates serum glycoproteins which are elevated in wound healing (152). CD206 also helps to modulate the cytokine secretion by these M2 macrophage, increasing IL-10 (an anti-inflammatory cytokine) but decreasing IL-12 (a pro-inflammatory cytokine) (153). For the mannose receptor expression in macrophage, it looks like the correct function of the OXPHOS metabolism is necessary, owing to the suppression of this pathway to cause the decrease of the CD206 expression (154). Furthermore, UDP-GlcNAc is required for the adequate glycosylation of the CD206 receptor (154). Therefore, the alteration of the OXPHOS metabolism and UDP-GlcNAc, an intermediate of the Krebs cycle, could decrease the M2 phenotype or the switch from M2 into M1 phenotype, and MSCs could be participating in these events.

#### c) Autoimmunity

To maintain homeostasis, the immune system must have an adequate balance between pro-inflammatory and anti-inflammatory responses and also be able to distinguish the nonself (foreign) from the self. If the system fails to provide adequate control of the inflammatory response and/or attacks what it is supposed to protect, it can lead to the development of an autoimmune disease (155, 156). As macrophage play an important role in maintaining tissue homeostasis and in the initiation and regulation of inflammation, they can be considered key players in the pathogenesis of autoimmunity. Indeed, excessive or uncontrolled inflammation is thought to be a common symptom of over 80 autoimmune diseases known to date, including rheumatoid arthritis (RA), experimental autoimmune encephalomyelitis (EAE), multiple sclerosis (MS), Crohn’s disease (CD), inflammatory bowel disease (IBD), and autoimmune hepatitis (37).

Macrophage polarization processes play a key role in the regulation of the inflammatory responses, where dysregulation can lead to autoimmunity. However, it is unknown whether an imbalance in the pro-inflammatory/anti-inflammatory macrophage’s (**Figure 1A**) ratio is a cause or a consequence of the different pathogenic processes leading to disease. Under this context, it has been well determined that a reduction in M2 macrophage, and/or continuous activation of M1 macrophage, could be playing an important role in the development of harmful inflammation and autoimmunity (157). Also, M1 macrophage is known to possess a metabolic shift towards glycolysis, which involves an increase in the glucose uptake, the conversion from pyruvate to lactate, and the production of reactive oxygen species (ROS) caused by the lower activity of the electron transportation chain (17). Furthermore, the detrimental effects of ROS produced by M1 macrophage may also be involved both in the initiation and progression of autoimmune diseases (158). Therefore, an increase in the M1/M2 ratio plays an important role in the pathogenesis of autoimmunity. M1 macrophages show an enhanced glycolytic metabolism, which allows the secretion of pro-inflammatory mediators that can lead to the development of chronic inflammation in autoimmune diseases (159). On the other hand, M2 macrophages exhibit an OXPHOS metabolism with enhanced catabolic pathways such as FAO (160).

Previous studies have shown that MSC can modulate the state of polarization in macrophage where they can promote the anti-inflammatory macrophage phenotype (161, 162). As we mentioned before, Vasandan et al., showed that MSC reduced the expression of GLUT1 and HK2, and increased the expression of CPT1α, a rate-limiting enzyme for mitochondrial β-oxidation in M1 macrophage, indicating that MSC modulates the state of polarization and therefore the activity of macrophage by inducing metabolic shifts (83). This effect of MSC on macrophage could be harnessed as a potential treatment for autoimmune diseases, restoring and inducing an adequate M1/M2 balance. An increase in the levels of glycolysis is a known hallmark of immune cell activation including macrophage, which has led to numerous attempts to treat autoimmune diseases by targeting (inhibiting) the glycolytic metabolism (163). It has been demonstrated that when macrophage precursors are exposed to insulin-like growth factor 2 (IGF-2) produced by MSC, it promotes an increase in the activity of the mitochondrial complex V, indicating a commitment toward OXPHOS metabolism (164). In turn, MSCs-produced IGF-2 induces an anti-inflammatory macrophage’s phenotype, which has been associated with ameliorating responses in the EAE model (165, 166). It has also been reported that MSC reprograms macrophage metabolism by increasing its lipid droplet biogenesis and PGE_2_ production (83). This metabolic MSCs-driven shift on macrophage is mediated through mTOR and PPARγ dependent pathways, proven to have therapeutic effects in a model of IBD (85). MSC-derived PGE_2_ interacts with the EP4 receptor in macrophage and therefore increases the release of anti-inflammatory cytokine IL-10. The soluble factors released by MSC, including IL-10, induce an anti-inflammatory macrophage phenotype through the activation of transcription factor STAT3 (167). STAT3 has been shown to up-regulate the DNA-damage-inducible transcript 4 (DDIT4), which is a TORC1 inhibitor. Therefore, STAT3 could act by inhibiting the transition from OXPHOS into glycolysis. In summary, the inhibition of TORC1 mediated by the upregulation of STAT3 with a glycolytic metabolism (**Figure 2**), reveal their importance in the polarization of the anti-inflammatory macrophage (**Figure 1B**) (168, 169), which could help in the treatment of autoimmune central nervous system (CNS) autoimmune diseases such as EAE and MS (170). For example, it has been reported that MSC-induced macrophages could exert powerful immunosuppressive and anti-inflammatory activity by inducing Treg cells and suppressing pro-inflammatory Th1 lymphocytes (171, 172). MSC-induced macrophages increase the secretion of amphiregulin (AREG), which is essential for the induction of Treg and suppress immune responses (171)

#### d) Regeneration

Regeneration is a process allowing the replacement of damaged tissue in terms of mass structure and function. It differs from the repair process which results in the formation of a scar with a collagen deposit and without the recovery of the functionality in the original tissue (173–175). In the early stages of embryonic development, mammals can regenerate certain tissues, organs as well as entire limbs. However, this capacity declines progressively during the development (176). In adulthood, the regenerative capacity of mammalian tissues and organs is very limited for most of them. Only a few tissues including the liver, pulmonary epithelium or even skeletal muscle retain the capacity to regenerate (177, 178).

During tissue regeneration in mammals and vertebrates, the presence and pivotal role of macrophage has been described throughout all processes (81, 179). Indeed, they are key players in the resolution of inflammation and tissue morphogenesis through the release of trophic factors (180). Macrophage depletion leads to the inhibition of regeneration (181–183). The polarization of macrophage, from a pro- to non-inflammatory phenotype, is also a key step to restore the homeostasis of the damaged tissue. Recruited within the first minutes after tissue injury or amputation, pro-inflammatory macrophage participate in the clearance of dead cells. Non-inflammatory macrophages are essential for the recruitment of new progenitor cells and the resolution of inflammation. The different subpopulations of macrophage are recruited sequentially according to a tight regulatory process and any alteration of this well-orchestrated recruitment leads to deleterious effects in tissue regeneration (181, 182, 184). Thus, understanding the mechanisms underlying the sequential recruitment and activation of macrophage in mammalian tissues able to regenerate and during tissue regeneration in regenerative vertebrates such as zebrafish is of great interest in the field of regenerative medicine (174–176, 181).

Skeletal muscle is an interesting model of regeneration to study and understand the role of the macrophage response (185). This tissue can regenerate after moderate injury and form new myofibers (186). Depletion of circulating monocytes in muscle, *via* the use of liposomes encapsulating clodronate, showed significant regeneration defects (183). Shortly after an injury, the majority of pro-inflammatory macrophage, positive for Ly6C, are recruited and start to express some cytokines such as TNF-α and IL-1β. These macrophage are mainly found when the necrotic myofibers are phagocytized by the macrophage, and when the muscle stem cells proliferate. Then, non-inflammatory macrophage that is negative for Ly6C and positive for IL-10 and TGF-β, are recruited rapidly after the injury, present during the whole regeneration process with a predominance at the end of the regeneration process when the small myofibers increase in size and the remodeling of the matrix takes place (187, 188). The alteration of this sequential recruitment of macrophage subpopulations tightly regulated during the regeneration process impairs muscle regeneration (189).

During regeneration, macrophage also responds to metabolic stimuli (185). Metabolic changes in macrophage occur before the modification of their inflammatory status. An increase in genes associated with the Krebs cycle and OXPHOS has been observed one or two days before the resolution of inflammation *in vitro* and could be under the influence of metabolic regulators such as AMPK (190, 191). Then, a decrease in the expression of glycolytic genes appears in macrophage subsets. This metabolic transition from glycolysis to OXPHOS could promote *in vivo* 1) macrophage polarization towards a non-inflammatory phenotype, 2) the resolution of inflammation, and 3) the regeneration of damaged muscle (185).

MSCs have received a lot of attention in regenerative medicine in recent years in part due to their paracrine functions and their immunosuppressive potential (192–194). As described above, they can modulate the metabolism of macrophage and their inflammatory status by promoting polarization towards a non-inflammatory phenotype, especially during wound healing (195). The use of MSC in regenerative therapy, although extremely promising, has not yet shown a clear improvement/restoration of tissue functions when administered in preclinical and clinical models of degenerative diseases such as osteoarthritis. Although, while MSC anti-inflammatory properties have been demonstrated *via* their capacity to regulate the pathological immune response *in vivo* resulting in short-term beneficial effects, their pro-regenerative potential that could lead to long-term beneficial effects has not yet been demonstrated (196). Indeed, intravenous injection of ASCs in an arthritic mouse model shows a decrease in inflammation and arthritis score. The study of the distribution of ASCs describes that they do not migrate into the joints but remain localized in the lung. Moreover, at 10 days after their injection, the cells were no longer detectable in the tissues. Therefore, they could act at distance *via* the secretion of factors, and thus potentially activate other cells such as macrophage towards repairing phenotype in a short period (197). It would be interesting to enhance MSCs to improve their survival and their pro-regenerative effect. Regarding what we have described in this review on the MSC/macrophage dialogue, modified MSC could communicate with macrophage to switch their metabolism and thus their inflammatory status by directing them towards a pro-regenerative phenotype and indirectly promoting regeneration.

## Conclusion

The metabolic regulation of macrophage is critical for their phenotype and function. In this context, MSC can induce a metabolic-associated anti-inflammatory phenotype to improve several biological processes including wound healing, immune tolerance, and regeneration. However, there is still little information regarding the specific mechanisms by which MSC can modulate the metabolism of macrophages to improve several diseases. Therefore. to unravel all these mechanisms will finally bring more information to ensure the safe clinical use of MSC on clinics and also to develop new strategies to improve their therapeutic properties.

## Author Contributions

NL-C and FB-B wrote the main part of the manuscript with input of CP, MA, CG, CB, FD and the supervision of AMV-L and PL-C. FD, RC-L, and RE-V critically revised the manuscript. All authors contributed to the article and approved the submitted version.

## Funding

This work was supported by grants from the Chilean National Commission for Scientific and Technological Investigation-CONICYT for National Agency of Investigation and Develop ANID: “Fondecyt Iniciación” N°11190690; “Fondecyt Regular” N°1211353 “Programa de apoyo a la formación de redes internacionales” N°180211; “Programa de Cooperación Científica ECOS-CONICYT” N°PC18S04-ECOS180032 and “Beca Doctorado Nacional” 2019 NL-C N° 2191997. We also acknowledge the Agence Nationale pour la Recherche (ANR) for the financial support with the project “PPAROA” (ANR-18-CE18-0010-02), Inserm and the University of Montpellier.

## Conflict of Interest

The authors declare that the research was conducted in the absence of any commercial or financial relationships that could be construed as a potential conflict of interest.
